# Ni(OH)_2_ and NiO Based Composites: Battery Type Electrode Materials for Hybrid Supercapacitor Devices

**DOI:** 10.3390/ma11071178

**Published:** 2018-07-10

**Authors:** Anne-Lise Brisse, Philippe Stevens, Gwenaëlle Toussaint, Olivier Crosnier, Thierry Brousse

**Affiliations:** 1 Department Electric Equipment Laboratory (LME), EDF R&D, Avenue des Renardières, 77818 Morêt-sur-Loing CEDEX, France; anne-lise.brisse@cnrs-imn.fr (A.-L.B.); philippe.stevens@edf.fr (P.S.); gwenaelle.toussaint@edf.fr (G.T.); 2 Institut des Matériaux Jean Rouxel (IMN), Université de Nantes, UMR CNRS 6502, 2 rue de la Houssinière BP32229, 44322 Nantes CEDEX 3, France; olivier.crosnier@univ-nantes.fr; 3 Réseau sur le Stockage Electrochimique de l’Energie, FR CNRS no. 3459, 80039 Amiens CEDEX, France

**Keywords:** electrochemical capacitors, nickel hydroxide, nickel oxide, hybrid device

## Abstract

Nanocomposites of Ni(OH)_2_ or NiO have successfully been used in electrodes in the last five years, but they have been falsely presented as pseudocapacitive electrodes for electrochemical capacitors and hybrid devices. Indeed, these nickel oxide or hydroxide electrodes are pure battery-type electrodes which store charges through faradaic processes as can be shown by cyclic voltammograms or constant current galvanostatic charge/discharge plots. Despite this misunderstanding, such electrodes can be of interest as positive electrodes in hybrid supercapacitors operating under KOH electrolyte, together with an activated carbon-negative electrode. This study indicates the requirements for the implementation of Ni(OH)_2_-based electrodes in hybrid designs and the improvements that are necessary in order to increase the energy and power densities of such devices. Mass loading is the key parameter which must be above 10 mg·cm^−2^ to correctly evaluate the performance of Ni(OH)_2_ or NiO-based nanocomposite electrodes and provide gravimetric capacity values. With such loadings, rate capability, capacity, cycling ability, energy and power densities can be accurately evaluated. Among the 80 papers analyzed in this study, there are indications that such nanocomposite electrode can successfully improve the performance of standard Ni(OH)_2_ (+)//6 M KOH//activated carbon (−) hybrid supercapacitor.

## 1. Introduction

The development of clean and sustainable energies is accompanied by a growing need for electrical energy storage devices. This is the reason batteries and electrochemical capacitors are so widely investigated by the scientific community. However, alone, neither of these devices can fulfill by themselves the requirements for power and energy densities required by some applications. The concept of hybrid supercapacitors has therefore been proposed. A hybrid supercapacitor combines a capacitive electrode and a faradaic electrode operating in either aqueous [1] or organic-based electrolytes [2]. Driven by the concern for increased safety, aqueous-based hybrid supercapacitors have attracted much interest over the last two decades. Towards the end of the 90s, most of the studies have focused on hybrid aqueous devices combining a capacitive negative electrode (typically activated carbon) and a faradaic positive electrode (Ni(OH)_2_, PbO_2_, …) [3]. Indeed, the use of a battery-type electrode as the positive electrode enables in some cases higher cell voltages compared to standard symmetric carbon//carbon EDLC operated in acidic or alkaline media. The maximum cell voltage is determined by the electrochemical stability window of the electrolyte, which for aqueous electrolytes is limited by the oxygen or hydrogen evolution reaction. Some battery-type positive electrodes exhibit high oxygen evolution overpotentials, which enables the electrode to operate above its thermodynamic potential limit. This resulting increase in cell voltage boosts the specific performance of the hybrid cell. In addition, the cell voltage increase is accompanied by an increase in specific capacity of the hybrid device. The explanation for this last phenomenon is due to the narrow electrochemical window in which the faradaic electrode is operated. The limited potential window of operation of the positive electrode enables the activated carbon-negative electrode to be operated in a much larger potential window compared to a symmetrical EDLC design in the same electrolyte. Since the capacitance (*C* in F·g^−1^) of the carbon electrode is the same as in a standard EDLC and since the potential window is increased (∆*U* in V), the resulting capacity (*Q* in C·g^−1^) is concomitantly increased [1,3,4] according to the well-known equation:

(1) Q=C×ΔU

Moreover, since the gravimetric capacity of the battery-type electrode material is much higher than that of the capacitive electrode, the mass of active material required for the positive (battery-type) electrode to balance the charge stored at the negative electrode (capacitive) is less than that required in a symmetrical design using two carbon-based capacitive electrodes. To fully understand the mechanisms taking place in such devices, some electrochemical concepts should first be detailed:

1. **Capacitive storage** implies an electrostatic surface reaction via the capacitive adsorption of ions in the electrochemical double layer. Indeed, when a potential is applied at the electrode surface, the charge accumulated in the solid is compensated by an electrolyte ion adsorption. The amount of charge depends of the applied potential and the stored energy varies linearly as a function of the potential. In this configuration, the ability to store energy is characterized by its capacitance and is expressed in Farads, i.e., C·V^−1^ (Equation (1)), or F. It must be pointed out that the value of the capacitance must remain constant all over the given potential window for ideal capacitive storage. The capacitance unit can be used to easily compare various capacitors, but such value always needs to be accompanied with the corresponding cell voltage in which the capacitor can be safely operated [5]. The same goes for a single electrode: the capacitance value measured in a three-electrode cell needs to be quoted with the potential window in which the electrode can be safely used to store charge.
2. **Faradaic charge storage** involves redox processes which imply an electronic transfer between the electrode and the electrolyte ions, thus leading to a change of oxidation state within the bulk of the electrode. The stored energy occurs at a quasi-constant potential and the charge stored depends of the quantity of electrons exchanged during the reaction. In this configuration, the stored charge (*Q*) is known as capacity and is expressed in C·g^−1^ or most commonly in mAh·g^−1^ in order to compare various types of batteries (Equation (2)).
  (2) $$Q\;\left(C\cdot g^{-1}\right)=\;\frac{n\times F\left(C\cdot {\mathrm{mol}}^{-1}\right)}{M\;\left(g\cdot {\mathrm{mol}}^{-1}\right)}$$
  where *n* is the number of exchanged electron, *F* the Faraday constant and *M* the molar mass of the reactant species.
3. **Pseudocapacitive** electrodes, such as those made of RuO_2_ or MnO_2_, store energy via fast redox processes limited to the electrode surface [6,7] but they have the same electrochemical signature than that of a capacitive electrode, i.e., exhibiting a linear dependence of the charge stored with voltage within the potential window range. Thus, a constant capacitance value is often provided to compare pseudocapacitive materials.

Many authors are claiming pseudocapacitive properties for a large range of materials that are commonly known as battery-type electrodes, some of which have been studied over more than 4 decades. This is especially true for Ni(OH)_2_ [8,9] which has been commercially used for many years as the positive electrode in Nickel-Zinc batteries, Nickel-Cadmium batteries, and Nickel-Metal Hydride batteries for examples. The confusion lies in the fact that due to the nanostructure or the nanocomposite structure of recently synthesized Ni(OH)_2_-based electrodes, they can be operated with much higher cycling rates than conventional Ni(OH)_2_ electrodes. However, even at such fast cycling rates a faradaic behavior of the electrode is observed, and it is virtually impossible to calculate a constant value that can be assimilated to a capacitance for such electrode. Unfortunately, there are a large number of reports in the literature for which such a calculation is still performed and a capacitance (in F) is given instead of the capacity (in mAh) of the electrode which is the correct parameter to be used [5,10].

**Figure 1:**
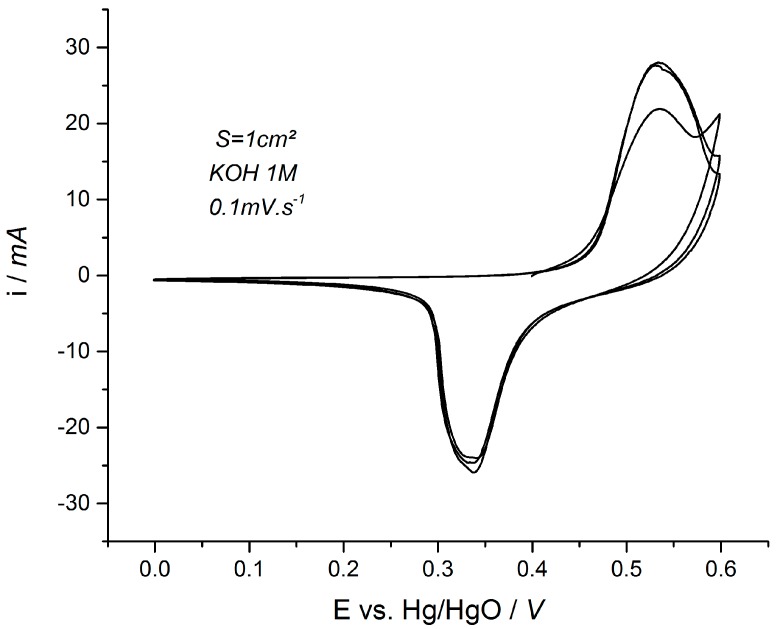
Typical representation of a cyclic voltammogram of a pure Ni(OH)_2_ electrode at a scan rate of 0.1 mV·s^−1^ in 1 M KOH electrolyte. Cycling was performed with a commercial Ni(OH)_2_ electrode for the purpose of this review.

This assumption is clearly illustrated in Figure 1 which shows the typical cyclic voltammogram (CV) of a Ni(OH)_2_ electrode in alkaline electrolyte. Integrating the oxidative sweep of the CV from 0.00 to 0.25 V vs. Hg/HgO leads to a capacity that is negligible (almost 0 C·g^−1^), which obviously translates to a capacitance of 0 F·g^−1^ if one tries to make the calculation. If the same calculation is done in a different potential window (0.45 to 0.60 V vs. Hg/HgO), the capacity is close to 40 C·g^−1^ but also a capacitance of 240 F·g^−1^ can be calculated by misguided authors. These calculations clearly show that the calculated “capacitance” for the Ni(OH)_2_ electrode is definitely not constant over the whole potential window. Consequently, Ni(OH)_2_ electrodes cannot be considered as pseudocapacitive, and their performance must only be expressed as the charge stored, i.e., the capacity (C·g^−1^ or mAh·g^−1^). It should be noted that such electrodes can be integrated in a hybrid device using Ni(OH)_2_ as the positive faradaic electrode and activated carbon as the negative capacitive electrode. In this case, the carbon-negative electrode will be operated in the potential region [−0.9 V; 0.1 V] vs. Hg/HgO reference electrode. The resulting signature of such a hybrid device is “capacitive-like” and a constant capacitance can usually be calculated for such device, but again this has nothing to do with a “pseudocapacitive behavior” which is an electrochemical concept only valid for a single electrode, and not for two terminal devices for which only electrical parameters can be associated. The possible origin of the confusion is suspected to come from this difference between the electrochemical behavior of a single electrode and the electrical signature of a hybrid cell [10]. The design of full hybrid cells is out of the scope of the present paper and will not be treated. The papers from Zheng et al., Le Comte et al., as well as the handbook of Beguin et al. give a full explanation on the determination of the performance of hybrid devices (energy and power densities …) [1,3,11].

However, despite this misleading interpretation of Ni(OH)_2_ electrochemical behavior reported in the literature, the nanocomposites involving this material can be of interest when used in a hybrid supercapacitor design. The motivation of this study is to collect and compare the true performance of different Ni(OH)_2_-based electrodes and their use as a positive electrode in hybrid supercapacitors. The requirements for the implementation of such electrodes in a hybrid design will be clearly listed as well as the improvements that are necessary in order to increase the energy and power densities of Ni(OH)_2_-based hybrid devices.

## 2. Methods

In this current paper, 80 scientific papers describing new Ni(OH)_2_ or NiO-based electrodes for supercapacitor [12,13,14,15,16,17,18,19,20,21,22,23,24,25,26,27,28,29,30,31,32,33,34,35,36,37,38,39,40,41,42,43,44,45,46,47,48,49,50,51,52,53,54,55,56,57,58,59,60,61,62,63,64,65,66,67,68,69,70,71,72,73,74,75,76,77,78,79,80,81,82,83,84,85,86,87,88,89,90,91,92] have been analyzed. The most significant papers were selected from different high impact factor journals.

In order to make a review of the different performance that nano-architectured Ni(OH)_2_ or NiO-based electrodes can bring to hybrid supercapacitors, all the misleading capacitance data were back-treated to express their capacity in coulombs per gram of active material. The capacity can be calculated from the galvanostatic discharge curves as described in Equation (3):

(3) $$Q_{galvanostatic\;discharge}=\int I\;dt$$

where *Q* is the charge stored (expressed in C·g^−1^), *I* the discharge current (A·g^−1^) and *t* the discharge time (in s).

Alternatively, the capacity can also be calculated from cyclic voltammetry when no galvanostatic measurements were made. For this purpose, the capacity is calculated by integration of the area under the CV curve (Equation (4)),

(4) $$Q_{cyclic\;voltammetry}=\frac{\int IdV}{mv}$$

where *Q* is the charge stored (expressed in C·g^−1^), *I* the current on discharge (A·g^−1^), *V* the potential (V), *v* the scan rate (V·s^−1^) and *m* the mass of active material (g).

Appropriate care was taken to make sure that the calculated capacities are expressed in coulombs per gram of active material and not per grams of composite nor electrode.

## 3. Results and Discussion

In the selected studies, half of the papers deal with the use of Ni(OH)_2_ and half of the papers are using NiO as the faradaic material. They are mainly carbon/Ni(OH)_2_ or carbon/NiO composites with various added carbon sources: nanotubes, porous carbon, graphene, graphene oxide (reduced or not), carbon cloth or activated carbon. Only 3 papers are not referring to a carbon-based material, mainly when the Ni component is nickel oxide. Concerning the electrode composition, nickel (foam, grid or foil) was the predominant current collector used (60% of the studied papers). As usual in the supercapacitor electrode preparation, the active nanocomposite material was most often mixed with carbon black and a binder, to form a rolled-pasted electrode or a slurry. It can be noted that this questions the use and the role of carbon in the preparation of the nanocomposite since carbon black is added to the electrode formulation anyway. Thus, the use of carbon additives to promote the electronic conductivity and concomitantly the power density of nanocomposite electrodes must be evaluated without using any other conductive additive in the electrode formulations. For one third of the selected papers the composite compound was used as prepared as a working electrode. For these electrodes, the composite was directly synthesized onto a nickel substrate, and they do not contain any binder nor conductive additives.

Capacities, calculated from galvanostatic discharge or cyclic voltammetry, were compared to the theoretical capacity of pure Ni(OH)_2_ (1041 C·g^−1^) or NiO (1292 C·g^−1^) to express a relative capacity in % of the theoretical capacity (Equation (5)).

(5) Qrelative=QelectrodeQtheoretical×100

**Figure 2:**
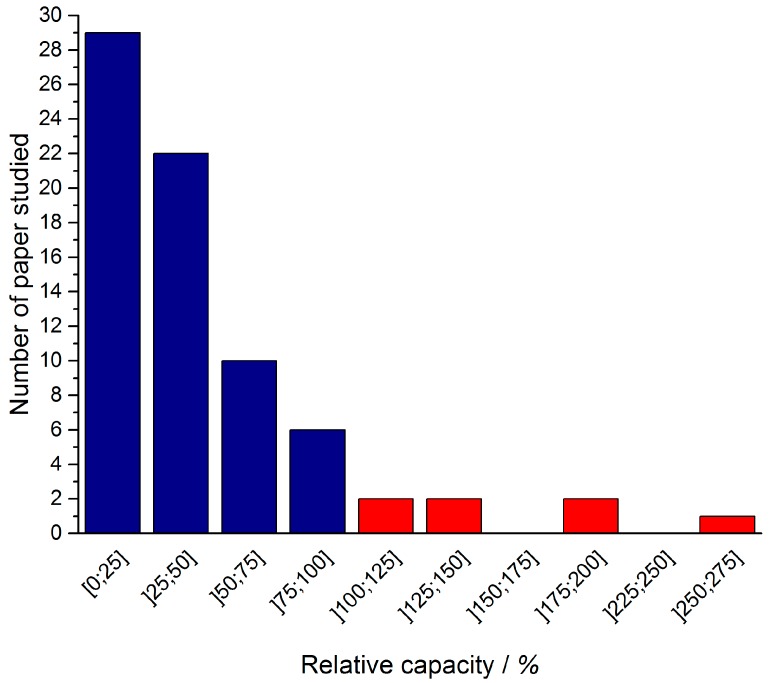
Relative capacity distribution upon the 80 electrode materials studied. The blue bars stand for electrode materials which exhibit less than 100% of the theoretical capacity and the red bars for those which exhibit relative capacity higher than 100%.

Of the 80 electrode materials studied: 51 exhibit relative capacities below 50%, 16 between 50% and 100%, and 7 above 100% (Figure 2). For 6 papers, it was not possible to re-express the capacity in coulombs per gram of active material due to the lack of information either on the electrode composition, or on the mass loading (which should have been expressed in mg·cm^−2^), or on the electrode area, …. These first observations are good indications that whatever the nano-architectures of Ni(OH)_2_-based faradaic electrodes, only a few of them have a capacity close to theoretical. For the purpose of clarity, we have arbitrarily chosen to show literature data reporting charging/discharging times less than 1000 s which correspond to a 3.6 C cycling rate. This choice will be explained later on in the related paragraph.

### 3.1. Litterature Findings

To compare the electrode performance reported in the literature for Ni(OH)_2_-based electrodes, we used a Ni(OH)_2_ sintered commercial battery electrode as a standard. To allow a fair comparison, all the capacities were related to the active material loading in the electrode. Indeed, the energy density of the electrode is not representative of the probable overall energy density of a full system. This is due to the presence of additional masses in the device such as the binder, the packaging material, the electrolyte, the current collectors, …. The active material weight accounts for about 33% of the total mass of the packaged commercial device and dividing by a factor of 3 is frequently used to extrapolate the energy density or power of the device from the performance of the material [11]. As explained by Gogotsi and Simon, the performance of a full system can be estimated only if the active material electrode is shaped in similar conditions to commercial electrode (100 to 200 µm thick and a loading above 10 mg·cm^−2^) [93]. When a 10 times thinner electrode is used to test a material for example, the gravimetric energy density would be equivalent to that of a 10 µm electrode divided by three to four (from 5 down to 1.5 Wh·kg^−1^ based on the cell weight), with only a slight increase in power density. Therefore, in order to fully compare the performance of different electrodes, their mass loading is a key parameter. Unfortunately, only 53% of the papers analyzed in this study indicate such loading (42 papers). The median of expressed loading is about 2 mg·cm^−2^, the minimum is 0.008 mg·cm^−2^ and the maximum 12 mg·cm^−2^. Therefore, only in a few cases can an extrapolation to cell performance be performed based on the reported mass loadings. Unfortunately, most of the papers still report energy and power densities despite inappropriate mass loadings, and as a consequence, the “outstanding” reported values are far beyond the reality which is obviously misleading for the readers.

**Figure 3:**
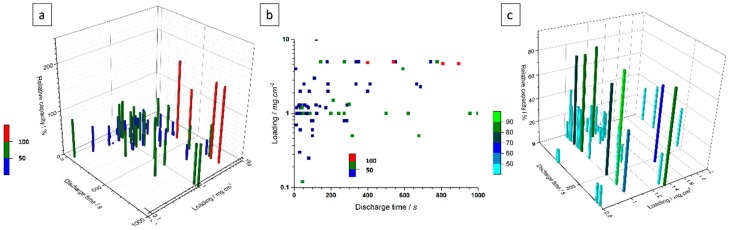
(**a**) 3D histogram of the performance of 42 different materials, showing their relative capacities, their discharge time and their loading; (**b**) Distribution of these materials in a 2D base with discharge time in abscissa and the loading in ordinate; (**c**) Zoom at 1 mg·cm^−2^ loading.

The different electrodes studied showing their relative capacities, their discharge time and their loading (in mg·cm^−2^) are compared in Figure 3. The maximum discharge times extracted from the literature data were arbitrarily limited to 1000 s since it seems to us that a discharge time of 17 min is closer to what is expected from a battery rather than from an electrochemical capacitor and that longer discharge times were not indicative at all of how fast the electrode kinetic can be. Indeed, to be implemented in a hybrid device Ni(OH)_2_ or NiO-based electrodes need to be able to undergo high cycling rates compatible with electrochemical capacitor applications (Figure 3a). Each bar has a color which refers to their relative capacity: below 50% of relative capacity (blue), between 50% and 100% (green) and above 100% (red). Obviously, there should not be any point above 100%, since this will mean that more than one electron is involved into the oxidation of Ni^2+^ to Ni^3+^. A capacity above 100%, i.e., higher than the theoretical capacity of NiO or Ni(OH)_2_, probably means that side reactions are occurring, thus providing unexpected extra capacity. This extra capacity as well as the existence of side reactions are rarely noted nor discussed by the authors. However, the capacities related to such side reactions are not removed when reporting the electrode capacity relative to wt. % of NiO or Ni(OH)_2_ which leads to an over-estimation of the capacity of the electroactive nickel compound. Alternatively, a capacity above 100% can be assigned to the inaccuracy in determining the active electrode mass. If this were the case, it might be expected that electrodes with low mass loadings, i.e., for which the accuracy on the mass loading determination would not be very high, would preferentially exhibit relative capacities above 100% due to underestimation of their mass. Interestingly, the four red bars (Figure 3a) are obtained with relatively high mass loadings for which the accuracy in mass determination should be quite reasonable. Thus, for such electrodes it is difficult to attribute an underestimation of the mass loading to the observed overcapacity and other explanations must be found. One possible explanation could be the presence of side reactions that provide extra capacity to the electrode. One typical example of side reactions is the oxidation of the nickel current collector when a too large surface of this collector is exposed to the electrolyte. For the calculation of the capacity, only the mass of NiO or Ni(OH)_2_ were considered; the mass of the current collector involved in this reaction is not included. The authors have not considered the oxidation of the nickel current collector which brings an extra capacity to the electrode. Therefore, the overall capacity of the electrode would come from both the active material (NiO or Ni(OH)_2_) and the nickel current collector. However, the authors have divided this capacity value by the mass of active material which generates an overestimation of the gravimetric capacity of the electrode. Oxidation of the carbon matrix can also provide such extra-capacity in the same manner as described above. Lastly, the extra capacity could be due to capacitive or faradaic charge storage of carbon additives which have not been removed from the calculation by the authors when calculating C·g^−1^ of NiO or Ni(OH)_2_. It is difficult to discriminate between these possible side reactions since we do not have access to the original data. However, we strongly encourage the authors to carefully examine the shape of their electrochemical plots which can probably bring some clues to evaluate the nature and the influence of side reactions in such electrodes.

It can be seen in Figure 3b that most of the points are centered on 1 mg·cm^−2^ which is a low loading to reasonably express gravimetric values for capacity and further for energy and power densities as previously mentioned. Moreover, most of the electrodes have been operated with a discharge time of 300 s or less, which seems a reasonable timescale for hybrid devices. However, the majority of these electrodes have a relative capacity of less than 50% of theoretical. Such a drop in relative capacity occurs concomitantly with a decrease in the mass loading which enables faster cycling rates (shorter discharge time) but at the expense of the capacity. Among all the data analyzed and reported in Figure 3b, we only found 4 papers describing interesting properties especially with regards to electrode kinetics. These latter papers depict high capacities at fast discharge times with composite electrodes containing nano-sized Ni(OH)_2_ and carbon (graphene, oxidized or not, and/or carbon nanotubes) [12,13,14,15]. It appears that the good electronic conductivity of carbon along with the nanometric size of the Ni(OH)_2_ particles synergistically enhance the rate of faradaic storage. It seems that facile electron transport and accessibility of the Ni(OH)_2_ particles enable the composite material to cycle at high scan rates without losing too much energy. 

**Figure 4:**
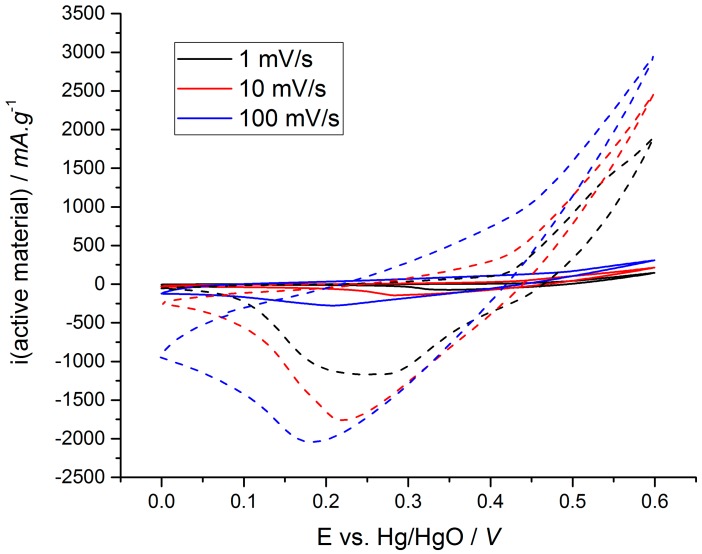
Cyclic voltammograms of Ni(OH)_2_ sintered electrode (straight line) and Ni(OH)_2_:carbon black composite electrode (dash line) in 1 M KOH electrolyte at 1, 10 and 100 mV·s^−1^.

Figure 3c also clearly shows that a significant number of electrodes exhibit a relative capacity above 20%. Indeed 20% of the theoretical capacity of Ni(OH)_2_ or NiO corresponds to about 200 C·g^−1^ which is a relatively high capacity for an activated carbon electrode in concentrated KOH electrolyte [94], and this at discharge times below 300 s. The observations depicted from Figure 3c, clearly show the interest of synthesizing a composite material combining carbon and Ni(OH)_2_ or NiO. These observations where confirmed in our laboratory as shown in Figure 4. Our own electrode material is made of carbon black and Ni(OH)_2_ synthesized by co-precipitation (details in Supplementary Materials). The solid line plots correspond to the commercial Ni(OH)_2_ sintered electrode whilst the dashed line is used for our Ni(OH)_2_:carbon black composite. The voltammograms do not show as well-defined oxidation peaks as those shown in Figure 1, thus suggesting that for scan rates higher than 0.1 mV·s^−1^ the electronic conductivity of the pure Ni(OH)_2_ is not sufficient to reveal fast oxidation reactions. However, the composite electrode displays higher capacities than the commercial electrode (pure Ni(OH)_2_) for all the scan rate from 1 to 100 mV·s^−1^. For example, at 1 mV·s^−1^ the Ni(OH)_2_ sintered electrode capacity is 223 C·g^−1^ (22% of the theoretical capacity) whereas the Ni(OH)_2_:carbon black composite electrode capacity is 620 C·g^−1^ (59% of the theoretical capacity).

Composite materials enable better electronic conductivity, as the carbon content plays the role of “superhighway” for electron released/consumed during the oxidation/reduction reaction. Thereby our composite material exhibits a capacity of 13 C·g^−1^ due to the redox processes at scan rate as high as 100 mV·s^−1^, corresponding to a full discharge in 6 s. Our own electrode was loaded to 10 mg·cm^−2^ compared to the 25 mg·cm^−2^ loading of the commercial electrode. At 1 mV·s^−1^ (i.e., 600 s of discharge time) scan rate, our results were in good agreement with those found in the literature for this loading (620 C·g^−1^, 59% of the theoretical capacity) [17,22,49,59,66].

All the results presented in Figure 3 and Figure 4 demonstrate that there might be some interest in nanocomposite Ni(OH)_2_-based electrodes as a positive electrode in hybrid supercapacitors, keeping in mind that the mass loading rarely exceeds a few mg·cm^−2^ for electrodes that keep a reasonable capacity at fast discharge rates. Moreover, we only report in Figure 3 the capacity of the active material, either Ni(OH)_2_ or NiO. The loading of active material in the nanocomposite electrodes we analyzed rarely exceeded 50% of the total electrode mass. Therefore, a capacity of 50% of theoretical obtained for a reasonable mass loading (5–8 mg·cm^−2^), translates in an electrode capacity close to 275 C·g^−1^, i.e., 50% × 50% × Q_th_ (Q_th_ = 1041 C·g^−1^ for Ni(OH)_2_ or 1292 C·g^−1^ for NiO). These high electrode capacities can be obtained at discharge times below 300 s. Such a performance cannot be achieved with the standard commercial electrode, thus indicating that nanocomposite Ni(OH)_2_-based electrodes can probably be implemented as a positive electrode in hybrid supercapacitors, but a consistent mass loading over 10 mg·cm^−2^ must be achieved. This can be a very practical goal for researchers working in that field. Indeed, carbon electrodes for commercial EDLC usually exhibit a thickness of 200 µm and a mass loading of 10 mg·cm^2^ [93], whilst a Ni(OH)_2_-based commercial electrode loading in standard Ni-MH battery ranges from 20 mg·cm^−2^ to 200 mg·cm^−2^ depending on the cell design: spirally wound, prismatic cell, pouch cell, … [95]. Such mass loadings can be achieved by standard electrode preparation processes such as tape casting, doctor blade or bar coating.

However, the capacity and rate capability are not the only important parameters for Ni(OH)_2_ or NiO-based electrodes, high cycling stability must also be achieved.

### 3.2. Cycling Ability of Ni(OH)_2_ Based Electrodes

**Figure 5:**
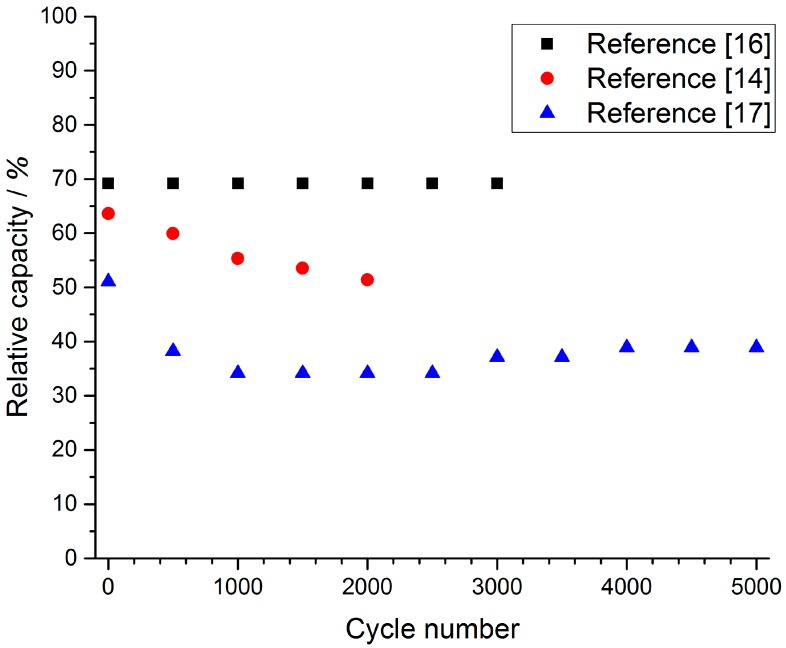
Evolution of the relative capacity of 3 different composite electrodes *vs.* cycle number cycled at 10 A·g^−1^ rate [14,16,17].

Assuming a daily cycle, a 20-year cycle life translates into at least 7000 cycles. More power demanding applications can probably ask for 10 times more cycles. The cyclability of the different nanocomposite electrode materials is therefore another key parameter that was reviewed. For 75% of the papers we analyzed, the authors also studied the cyclability of their electrodes, for the remaining 25% it was just not studied at all or studied through a full device with a design that is often questionable from the point of view of the mass balance of each electrode but also from the choice of operating cell voltage, the choice of the negative electrode, and the testing parameters. Only the cycling performance of single Ni(OH)_2_ or NiO-based electrodes in half cell configuration are therefore reported here. The number of cycles was different from one paper to another, with a range from 500 [57] to 30,000 cycles [70], but most of the reported cycle numbers ranged from 1000 to 2000 cycles. The comparison of these different electrode material performances was difficult because the authors have chosen very disparate current densities (from 1 to 28.6 A·g^−1^). In a few studies, the cycling performance was evaluated using cyclic voltammetry experiments with scan rates between 50 and 100 mV·s^−1^. Galvanostatic cycling tests were performed at 1 A·g^−1^ [30,37,54,60,82], 5 A·g^−1^ [44,49,67,89] and 10 A·g^−1^ [14,16,17], as they were the most recurring values of current density. We have chosen to report the results for 10 A·g^−1^ current density in Figure 5, which shows the relative capacity (% of theoretical capacity as previously detailed) as a function of the number of cycles. Indeed, capacities for the other current densities were not of interest as they were below 40% of the theoretical capacity. Relative capacities for these cycling tests were calculated based on the potential range used for the cyclic voltammetry tests.

Different cycling stabilities were observed among the materials studied, but it could be seen that for materials which display high relative capacities, this capacity remains relatively stable, even over 2000 cycles (Figure 5). It is noteworthy that the discharge time for these cycling performance tests ranges between 40 and 70 s, which is compatible with hybrid supercapacitor applications. However, longer cycling tests need to be performed in order to validate the value of nanocomposite Ni(OH)_2_ or NiO-based electrodes in hybrid devices. Once again, we suggest that a reasonable mass loading must be used (above 10 mg·cm^−2^), and the ability to do over 10,000 cycles for a single electrode and full devices using activated carbon as negative electrode must also be investigated. Today, the cycling ability of nanocomposite positive electrode is not clearly established.

## 4. Conclusions

Nanocomposite Ni(OH)_2_ or NiO-based electrodes can be interesting positive faradaic electrode materials for aqueous hybrid devices. It is important to keep in mind that nickel hydroxide will remain a battery-type electrode, and not a pseudocapacitive material. Capacities (and not capacitances) therefore need to be expressed in C·g^−1^ or mAh·g^−1^. Indeed C·g^−1^ or mAh·g^−1^ is more appropriate to compare battery-type materials, but also for supercapacitor-type materials when they contain bi-material composite electrodes having some element of faradaic storage mechanisms [96]. This study points out the strengths and drawbacks of nanocomposite Ni(OH)_2_ or NiO-based electrodes reported in the literature. The requirements for these electrodes are much more drastic than for standard Ni(OH)_2_ used in secondary batteries such as Nickel-Metal hydride, Nickel-Zinc, .... Only a few reported electrodes effectively meet the requirements for implementation as positive faradaic electrode in hybrid supercapacitors.

1. Firstly, the preparation routes are often more complex than standard Ni(OH)_2_ battery-type electrodes, unlike what most of the authors are claiming.
2. Secondly, only a few studies are using reasonable mass loadings (≥10 mg·cm^−2^) which are necessary to validate the gravimetric values reported for capacity, energy and power densities. There is a need to investigate electrodes with such reasonable loadings, especially with regards to the capacity and the rate capability that can be achieved in a real-life hybrid device. The use of standard electrode preparation processes such as tape casting, doctor blade or bar coating will provide such mass loadings.
3. Thirdly, the cyclability is also a key parameter for Ni(OH)_2_ or NiO-based electrodes for supercapacitors application, and high cycling stability (≥7000 cycles) must be demonstrated depending upon the targeted application. Again, a sufficient mass loading must be used for these studies. Moreover, this parameter has to be evaluated not only for a single electrode but also when implemented in optimized full devices.

Finally, the performance of hybrid devices using such electrodes must be investigated in reasonable size designs involving at least a few cm^2^ electrodes. Contemporary literature data indicates that nanocomposite Ni(OH)_2_ or NiO-based electrodes could fulfill the above-mentioned requirements but there is still no clear study corroborating this assumption. These three prerequisites should serve as a guideline for future investigations of future nanocomposite electrodes design to be used as faradaic electrode of hybrid supercapacitors.

## Supplementary Materials

The following are available online at http://www.mdpi.com/1996-1944/11/7/1178/s1, Ni(OH)_2_: carbon black synthesis and characterization details.
