# Supplementary material for: Ni(OH)2 and NiO Based Composites: Battery Type Electrode Materials for Hybrid Supercapacitor Devices
Source: Materials (Basel). 2018 Jul 10;11(7):1178. doi: 10.3390/ma11071178 (PMC6073142; doi:10.3390/ma11071178)
Supplement: Supplementary file 1 [file materials-11-01178-s001.pdf]

Supporting information

1. Materials

Carbon black >99% was acquired from Superior Graphite Co. Analytical grade $Ni(NO_3)_2.6H_2O$, sulfuric acid, nitric acid and KOH were purchased from Alfa Aesar, and were used without further purification.

2. $Ni(OH)_2$: carbon black composite powder synthesis

$Ni(OH)_2$: carbon black composites were prepared by a precipitation method adapted from [1–3]. 30 mL of a solution of $Ni(NO_3)_2.6H_2O$ 0.1M was prepared, and a defined amount of carbon black was added to the solution in order to obtain a composite material containing 50%wt of carbon in the final product. The suspension was agitated 1 hour with ultrasonication. KOH 0.1M was then added drop by drop to produce a pH=8.5. The suspension was centrifuged at 8000 rpm for 10 min and washed several times in deionized water. The final product was then dried under vacuum in an oven at 120°C.

3. Characterization

In order to determine the nickel hydroxide / carbon ratio in the synthesized composite materials, thermogravimetry (TG) measurements were performed using a STA 449 F3 (Netzsch). Samples of typically 10 mg were placed in alumina crucibles and heated from 50°C to 1000°C at 1°C/min under an atmosphere of Ar/O_2 mixture (**Figure 1**). The pure cuprous oxide $Ni(OH)_2$, is oxidized to NiO at around 215°C. For the pure carbon black powder, a gradual mass loss can be observed from 600°C to 700°C, until complete oxidation of the carbon into CO_2. In the case of the composite powder, a loss of 65%wt of the total mass is observed, which corresponds to a 37.5/62.5 weight ratio of nickel hydroxide/carbon black in the composite powder.

The morphology of our composite powder (Figure 2) and of the commercial electrode (Figure 3) was investigated via SEM images. Figure 2 shows that the $Ni(OH)_2$ particles are embedded

in a carbon black matrix. From the backscattered electron images it can be seen that the $Ni(OH)_2$ particle size ranges from less than 1 μm to a few μm. These grains are surrounded by 100 nm diameter carbon black particles which provide a local conductive network which is definitely not present in the commercial electrode. For the $Ni(OH)_2$ commercial electrode, the powder scratched from the sintered electrode (Figure 3) clearly shows a large particle size distribution (10 to 100 μm) without any carbon black. Some particles are formed of platelet-like smaller crystals in the range of few tens of nm (Figure 3).

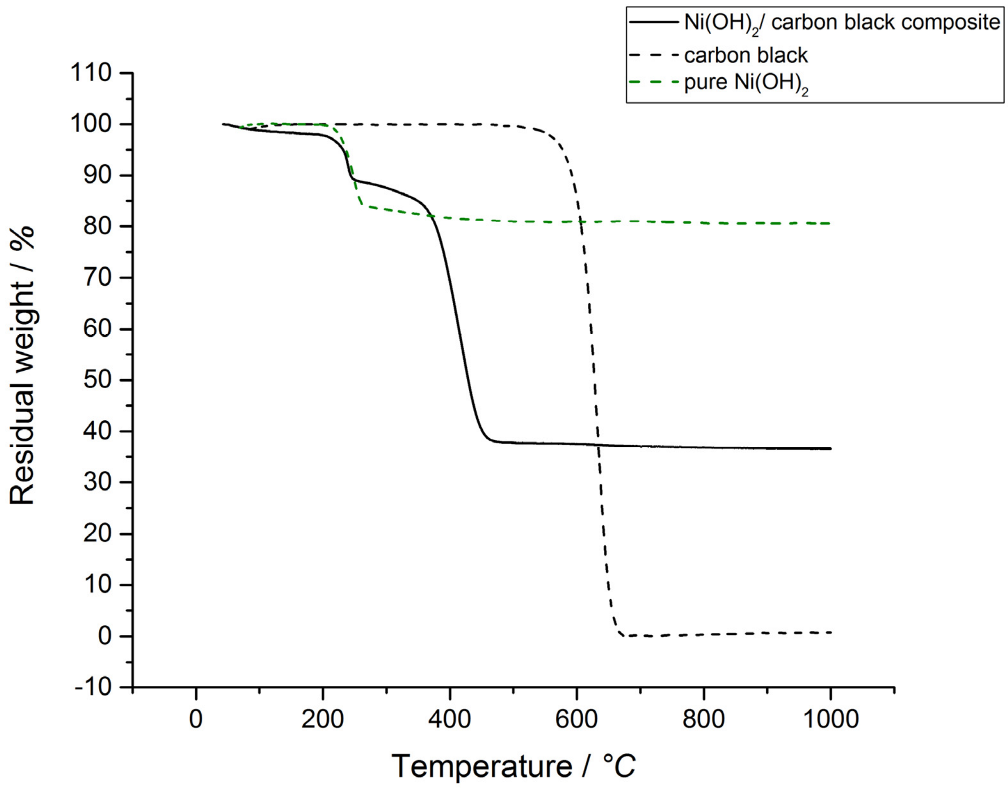

Figure 1: Thermogravimetric analysis of carbon black, pure $Ni(OH)_2$ and our composite material $Ni(OH)_2$/carbon black.

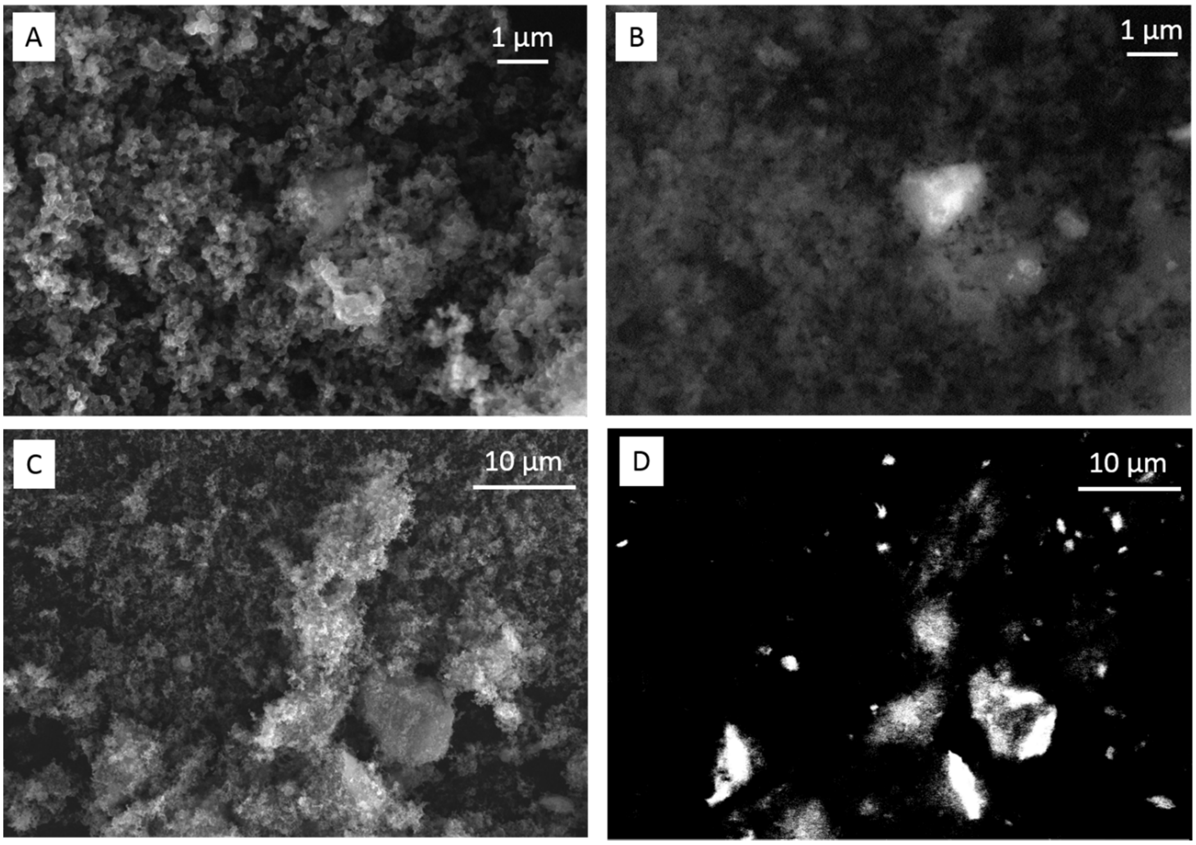

Figure 2: SEM images of the $Ni(OH)_2$/carbon black composite in secondary electron (A, C) and in backscatter electron (B, D).

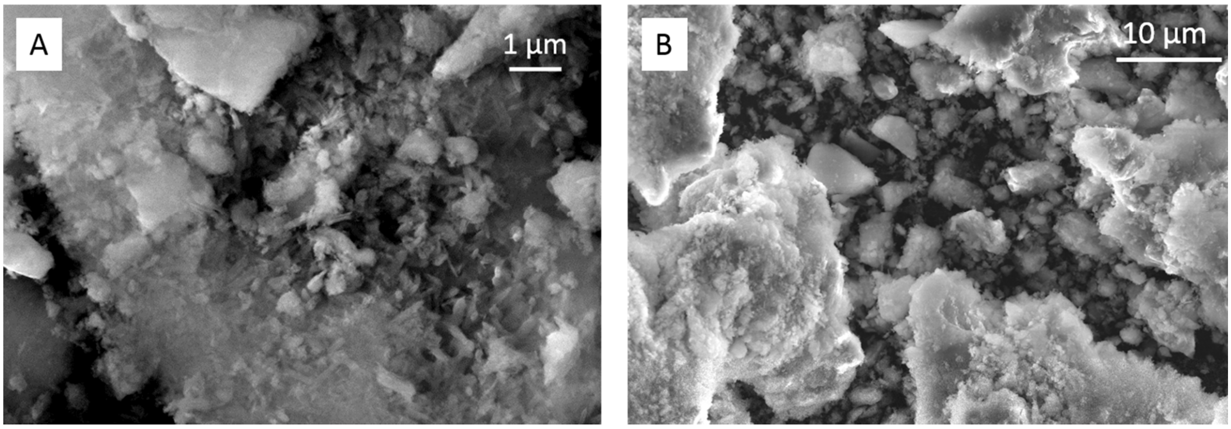

Figure 3: SEM image of the commercial electrode in secondary electron.

4. Electrochemical measurements

Composite electrodes were prepared by mixing the active material in ethanol with PTFE (60 wt % dispersion in H_2O, Sigma Aldrich) with a weight ratio of 90/10. The mixture was heated at 60-70°C to partially evaporate the solvent. The resulting black paste was then cold laminated into thick films (30-100 µm) that were subsequently dried at 60°C in air. Finally, 12 mm-diameter electrodes (corresponding to mass loadings of about 10 $mg.cm^{-2}$) were cut out from the films and pressed at 900 MPa for 1 minute on a stainless steel grid (316L, 60 mesh, 0.160 mm, Saulas).

Electrochemical experiments were performed in a 1M KOH aqueous electrolyte with a VMP3 galvanostat–potentiostat from Biologic operated under EC-Lab software. The three-electrode measurements were all conducted with an Hg/HgO/KOH (1M) reference electrode and a platinum grid counter electrode.

[1] J.Y. Lee, K. Liang, K.H. An, Y.H. Lee, Nickel oxide/carbon nanotubes nanocomposite for electrochemical capacitance, Synthetic Metals. 150 (2005) 153–157. doi:10.1016/j.synthmet.2005.01.016.
[2] Y. Wang, L. Yu, Y. Xia, Electrochemical Capacitance Performance of Hybrid Supercapacitors Based on Ni(OH)[sub 2]⁄Carbon Nanotube Composites and Activated Carbon, Journal of The Electrochemical Society. 153 (2006) A743. doi:10.1149/1.2171833.
[3] Q. Huang, X. Wang, J. Li, C. Dai, S. Gamboa, P.J. Sebastian, Nickel hydroxide/activated carbon composite electrodes for electrochemical capacitors, Journal of Power Sources. 164 (2007) 425–429. doi:10.1016/j.jpowsour.2006.09.066.
[4] K. Min, H. Gao, K. Matyjaszewski, Use of Ascorbic Acid as Reducing Agent for Synthesis of Well-Defined Polymers by ARGET ATRP, Macromolecules. 40 (2007) 1789–1791. doi:10.1021/ma0702041.
